# Measured body size and serum estrogen metabolism in postmenopausal women: the Ghana Breast Health Study

**DOI:** 10.1186/s13058-022-01500-8

**Published:** 2022-01-26

**Authors:** Ashley M. Geczik, Roni T. Falk, Xia Xu, Daniel Ansong, Joel Yarney, Beatrice Wiafe-Addai, Lawrence Edusei, Florence Dedey, Verna Vanderpuye, Nicholas Titiloye, Ernest Adjei, Francis Aitpillah, Ernest Osei-Bonsu, Joseph Oppong, Richard Biritwum, Kofi Nyarko, Seth Wiafe, Baffour Awuah, Joe-Nat Clegg-Lamptey, Thomas U. Ahearn, Jonine Figueroa, Montserrat Garcia-Closas, Louise A. Brinton, Britton Trabert

**Affiliations:** 1 Division of Cancer Epidemiology and Genetics, National Cancer Institute, National Institutes of Health (NIH), DHSS, 9609 Medical Center Dr., Bethesda, MD 20892 USA; 2 Protein Characterization Laboratory, Leidos-Frederick, Inc., Frederick National Laboratory for Cancer Research, Frederick, MD USA; 3 Kwame Nkrumah University of Science and Technology, Kumasi, Ghana; 4 Korle Bu Teaching Hospital, Accra, Ghana; 5 Peace and Love Hospital, Kumasi, Ghana; 6 Komfo Anokye Teaching Hospital, Kumasi, Ghana; 7 University of Ghana, Accra, Ghana; 8 Loma Linda University, School of Public Health, Loma Linda, CA USA; 9 The University of Edinburgh, Cancer Research UK Edinburgh Center, Edinburgh, Scotland

**Keywords:** Measured body mass index, Height, Waist-to-hip ratio, Estrogen metabolism, Postmenopausal Black women

## Abstract

**Background:**

Several anthropometric measures have been associated with hormone-related cancers, and it has been shown that estrogen metabolism in postmenopausal women plays an important role in these relationships. However, little is known about circulating estrogen levels in African women, and the relevance to breast cancer or breast cancer risk factors. To shed further light on the relationship of anthropometric factors and estrogen levels in African women, we examined whether measured body mass index (BMI), waist-to-hip ratio (WHR), height, and self-reported body size were associated with serum estrogens/estrogen metabolites in a cross-sectional analysis among postmenopausal population-based controls of the Ghana Breast Health Study.

**Methods:**

Fifteen estrogens/estrogen metabolites were quantified using liquid chromatography-tandem mass spectrometry in serum samples collected from postmenopausal female controls enrolled in the Ghana Breast Health Study, a population-based case–control study conducted in Accra and Kumasi. Geometric means (GMs) of estrogens/estrogen metabolites were estimated using linear regression, adjusting for potential confounders.

**Results:**

Measured BMI (≥ 30 vs. 18.5–24.9 kg/m^2^) was positively associated with parent estrogens (multivariable adjusted GM for unconjugated estrone: 78.90 (66.57–93.53) vs. 50.89 (43.47–59.59), *p*-value < 0.0001; and unconjugated estradiol: 27.83 (21.47–36.07) vs. 13.26 (10.37–16.95), *p*-value < 0.0001). Independent of unconjugated estradiol, measured BMI was associated with lower levels of 2-pathway metabolites and higher levels of 16-ketoestradriol. Similar patterns of association were found with WHR; however, the associations were not entirely independent of BMI. Height was not associated with postmenopausal estrogens/estrogen metabolite levels in African women.

**Conclusions:**

We observed strong associations between measured BMI and parent estrogens and estrogen metabolite patterns that largely mirrored relations that have previously been associated with higher breast cancer risk in postmenopausal White women. The consistency of the BMI-estrogen metabolism associations in our study with those previously noted among White women suggests that estrogens likely explain part of the BMI-postmenopausal breast cancer risk in both groups. These findings merit evaluation in Black women, including prospective studies.

**Supplementary Information:**

The online version contains supplementary material available at 10.1186/s13058-022-01500-8.

## Introduction

Anthropometric measures, such as body mass index (BMI) and height, are associated with increased postmenopausal breast cancer risk in studies conducted among predominantly non-Hispanic White women [1–3]. Recent meta-analyses have estimated 15% higher risk of postmenopausal breast cancer [2] in overweight or obese compared with lean women. Central adiposity measured by waist circumference (WC) or waist-to-hip ratio (WHR) has also shown strong positive associations with breast cancer risk [3, 4] although these are attenuated after accounting for BMI. One of the hypothesized mechanisms for these relations is that overweight and obese women have elevated levels of circulating estrogens [5, 6], as adipocytes produce estrogens from androgens via aromatase activity [7]. Height may indicate early life nutritional status and high levels of endogenous proliferative hormone such as estrogens. Adult height is also a risk factor for breast cancer, most notably hormone receptor positive tumors in both pre- and postmenopausal women [8].

In Westernized countries and based on predominantly White study populations, it has been established that estrogens are key hormones in breast carcinogenesis. Parent estrogens (estradiol and estrone) stimulate cell proliferation via estrogen receptor-mediated pathways. When parent estrogens are hydroxylated at one of three carbon positions of the steroid ring, metabolites are formed along three different pathways (i.e., 2-, 4-, and 16-hydroxylation pathways). The carcinogenicity of individual estrogen metabolites can vary. Catechol estrogen metabolites can stimulate cell proliferation via estrogen receptor-dependent pathways and induce DNA damage directly by forming quinone DNA adducts or indirectly via redox cycling [9]. Methylation of the catechol estrogen metabolites can prevent mutagenic quinone formation [10]. Prospective epidemiologic studies of breast cancer risk have suggested that metabolism favoring parent estrogens into the 2- and 4-pathway over the 16-pathway is associated with lower postmenopausal breast cancer risk [11].

Epidemiologic evidence supports that among women not using exogenous hormones, circulating levels of estrogens are higher in overweight and obese US women [1, 5, 6]. In a pooled analysis of eight studies, estradiol was 83% higher in postmenopausal obese women compared with lean women, and estrone was 60% higher [12]. In a study in the Women’s Health Initiative Observational Study, BMI was positively associated with circulating parent estrogens and reduced methylation of catechol estrogen metabolites [13]. These findings are consistent with the patterns associated with higher breast cancer risk [11], however, the study populations evaluated to date have predominantly included non-Hispanic White women.

Little is known about estrogen levels in African women, and the relevance to breast cancer or breast cancer risk factors. It has been reported previously that US Black women have higher circulating estradiol independent of adiposity and experience less reduction in levels with weight loss than White women [14]. Further, it has been hypothesized that these hormonal differences may contribute to differential patterns of breast cancer incidence and mortality by race [15, 16]. Thus, investigations to understand sex steroid hormone associations with anthropometric characteristics in African women will contribute to understanding breast cancer risk factors in this population. We had the opportunity to examine this using the population-based postmenopausal controls of the Ghana Breast Health Study conducted in two large metropolitan areas where obesity has been demonstrated to be a breast cancer risk factor [17].

## Materials and methods

### Study population

For the current cross-sectional analysis, we utilized data from postmenopausal female controls enrolled in the Ghana Breast Health Study, a multi-disciplinary population-based case–control study. The methodology of the original study is described in more detail elsewhere [17, 18]. In brief, population controls were selected on the basis of frequency matching to breast cancer cases (enrolled at Korle Bu Teaching Hospital in Accra and Komfo Anoyke Teaching Hospital and Peace and Love Hospital in Kumasi) on age, with similar restrictions regarding case catchment areas, and at least 1 year of residence in these areas. The 2011 Ghanaian census was used to select enumeration areas (areas comprised of ~ 750 residents) of the districts from which cases were expected to derive. Trained census workers enumerated all households with respect to the sex and age of the residents. When households were enumerated, a brochure was left explaining the study and encouraging participation should an individual be selected for inclusion. After selected areas had been enumerated, individuals were randomly selected to approximate the age distribution of female breast cancer cases expected during the study. Study personnel visited subjects’ homes to determine eligibility, inform them of study selection and invite them for a hospital visit.

Controls were approached for in-person interviews by trained personnel who recorded information on standardized questionnaires. Interviews were generally conducted in the hospitals, although a few were administered at the subjects’ homes. The interview response rate was 91.9%. Of 2106 potentially eligible controls, we excluded the following women who at interview indicated that they were premenopausal (*n* = 1237), did not know their menopause status (*n* = 9), reported current hormone use (*n* = 10), or could not provide information on current hormone use (*n* = 16). An additional 199 women were excluded because they did not have enough serum volume available for the estrogen/estrogen metabolite assays. Of the 635 samples from which estrogens were measured, an additional 3 were excluded because of missing age and 47 because of reports of menstrual bleeding on the blood draw questionnaire, suggesting that they were either perimenopausal or premenopausal. The final analytic population consisted of 585 postmenopausal controls that had information on at least one variable related to body size.

### Exposure assessment

**Fig. 1:**
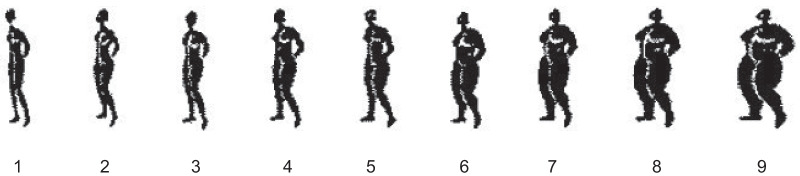
Body size silhouettes as shown in study questionnaire. Question asked respondents to indicate which silhouette best represented their current body shape

The study questionnaire focused on established breast cancer risk factors including demographic factors, menstrual and reproductive characteristics, family history of breast cancer, medical history, occupational history, and anthropometric and physical activity variables. Anthropometric measures included a 9-scale pictogram for participants to self-identify body shape (Fig. 1), with 1 corresponding to the slimmest body shape and 9 the heaviest body shape. Based on the self-reported body size silhouette scale (1–9), women were categorized for analyses as slight (1 or 2), average (3 or 4), slightly heavy (5 or 6), or heavy (7, 8, or 9). Weight, standing height, waist circumference, and hip circumference were measured by trained staff at an in-person interview. Measurements were made at least twice in the same setting and averaged. BMI (kg/m^2^) was calculated as weight (in kilograms) divided by height (in meters squared). WHR was calculated based on measured waist circumference (cm) divided by measured hip circumference (cm).

### Laboratory assays

Details of the hormone assay have been published previously [19–21]. Briefly, stable isotope dilution liquid chromatography-tandem mass spectrometry (LC–MS/MS) was used to quantify 15 estrogens and estrogen metabolites including: estrone, estradiol, 2-pathway metabolites (2-hydroxyestrone, 2-methoxyestrone, 2-hydroxyestradiol, 2-methoxyestradiol, and 2-hydroxyestrone-3-methyl ether); 4-pathway metabolites (4-hydroxyestrone, 4-methoxyestrone, and 4-methoxyestradiol); and 16α-pathway metabolites (16α-hydroxyestrone, estriol, 16-ketoestradiol, 16-epiestriol, and 17-epiestriol). This method detects 15 estrogens and estrogen metabolites in serum which circulate, at least in part, as sulfated and/or glucuronidated conjugates to facilitate storage, transport, and excretion. Five of the estrogens (estrone, estradiol, estriol, 2-methoxyestrone and 2-methoxyestradiol) were also measured in unconjugated forms in circulation. For those metabolites with both combined and unconjugated measurements, the concentration of the conjugated form was calculated as the difference between the combined estrogen measurement and the unconjugated estrogen measurement; for estradiol that calculation was (conjugated estradiol = combined estradiol – unconjugated estradiol). The limit of detection for each estrogen and estrogen metabolite measured using this LC–MS/MS assay was 10 fg on column (approximately 0.33–0.37 pmol/L) [19, 22]. There were no samples in the current study with undetectable levels for any of the hormones measured. Laboratory coefficients of variation (CV) of blinded quality control duplicates distributed within and across batches were < 5% for all hormones measured. Intraclass correlation coefficients (ICCs) ranged from 0.97 to 0.998 with a median value of 0.99.

### Statistical analysis

After log-transformation of data to improve normality, geometric means (GM) (pmol/L) of individual serum estrogens/estrogen metabolite concentration by exposure categories were estimated using linear regression adjusting for potential confounders: age at blood draw, blood draw year, smoking status (never, former, current, unknown/missing), time since menopause (≤ 2, 3–5, 6–10, > 10 years, missing), and oral contraceptive use (ever, never). We performed a test for trend by including the exposure in the model as an ordinal variable. The percent change (%Δ) in GMs between the highest and the lowest categories was estimated by taking the ratio of the GM difference between the two categories over the GM of the reference category, multiplied by 100. We statistically tested for the difference using a Wald test.

Several secondary analyses were performed. First, for BMI (and other measures of body size), we additionally adjusted for unconjugated estradiol to examine whether the associations with other estrogen metabolites were driven by their correlations with unconjugated estradiol, the estrogen most strongly correlated with measured BMI (Spearman *r* = 0.43). Next, we investigated whether BMI was associated with altered patterns of estrogen metabolism, using pathway groups. We compared the mean proportions of parent estrogens out of summed estrogens/estrogen metabolites across BMI categories with adjustment for the summed concentration of estrogens/estrogen metabolites. Further, because 2-, 4-, and 16-pathway metabolites (“child metabolites”) are metabolized from a limited pool of shared precursors (parent estrogens), an increase in the level of one downstream pathway indicates a reduction in levels of other competing pathways. To address this, we modeled proportions of each child metabolite pathway group (2-pathway metabolites: 2-catechols [2-hydroxyestrone, 2-hydroxyestradiol] and methylated 2-catechols [2-methoxyestrone and 2-methoxyestradiol]; 4-pathway metabolites: 4-catechols [4-hydroxyestrone] and methylated 4-catechols [4-methoxyestrone, 4-methoxyestradiol]; 16-pathway metabolites: [16α-hydroxyestrone, estriol, 16-ketoestradiol, 16-epiestriol, 17-epiestriol]) out of summed child metabolites, with adjustment for the summed concentration of child metabolites. This approach estimates the association with replacement of one pathway group for other pathway groups while holding summed child metabolites constant. We tested for any difference across BMI categories using global F test; if there was a significant difference (*p* < 0.05), we performed pairwise t-tests for three combinations of BMI comparisons (25–29.9 vs. 18.5–24.9 kg/m^2^; ≥ 30 vs. 18.5–24.9 kg/m^2^; ≥ 30 vs. 25–29.9 kg/m^2^) and six current body size comparisons (heavy vs. slight, slightly heavy vs. slight, average vs. slight, heavy vs. average, slightly heavy vs. average, and heavy vs. slightly heavy).

Finally, because underlying diseases may influence the associations, we performed sensitivity analyses after excluding women diagnosed with a history of diabetes (*n* = 49) and excluding women with low BMIs (< 18.5 kg/m^2^) (*n* = 20).

All statistical tests were two-sided with 5% type I error. *Q*-values reflecting the false discovery rates (FDR) were calculated to address multiple comparisons (25 tests per exposure) separately for the original model, the model with additional adjustment for unconjugated estradiol, and the model with additional adjustment for measured BMI (where applicable). Analyses were conducted with SAS version 9 (SAS Institute).

## Results

Among 585 postmenopausal African women, the average age at blood draw was 56.8 years (standard deviation 8.1 years) (Table 1). Most women reported never smoking (95.0%), not having a history of diabetes (87.9%), never using oral contraceptives (84.8%), and having given birth to at least 3 children (81.6%). The distribution of measured BMI categories among study participants was as follows: 3.4% underweight (< 18.5 kg/m^2^), 33.0% healthy weight (18.5- < 25.0 kg/m^2^), 28.9% overweight (25.0- < 30.0 kg/m^2^), and 26.3% obese (≥ 30.0 kg/m^2^); 8.4% of women had missing data on either measured height and/or weight.

**Table 1:** Distribution of demographic and measured anthropometric factors in Ghana Breast Health Study Postmenopausal Controls (*n* = 585)

	Mean	Standard Deviation
Age at blood draw	56.8	8.1
Age at menopause	48.3	5.1

	*N*	Percent
*Time since menopause*		
≤ 2	104	17.8
3–5	105	18.0
6–10	130	22.2
> 10	153	26.2
Missing	93	15.9
*Year of blood draw*		
2013	229	39.2
2014	193	33.0
2015	163	27.9
*Smoking status*		
Current	0	0.0
Former	4	0.7
Never	556	95.0
Unknown	5	0.9
Missing	20	3.4
*Diabetes*		
Ever	49	8.4
Never	514	87.9
Unknown	22	3.8
*Age at menarche*		
< 14	112	19.2
15	171	29.2
16	107	18.3
≥ 17	109	18.6
Unknown	86	14.7
*Parity/Number of births*		
Nulliparous	16	2.7
1–2	91	15.6
3–4	200	34.2
5 +	277	47.4
Unknown	1	0.2
*Oral contraceptive use*		
Ever	89	15.2
Never	496	84.8
Unknown	0	0.0
*Age at menopause*		
< 45	95	16.2
45–54	349	59.7
≥ 55	48	8.2
Unknown	93	15.9
*BMI (kg/m*^*2*^*)*		
Underweight (< 18.5)	20	3.4
Healthy weight (18.5–24.9)	193	33.0
Overweight (25.0–29.9)	169	28.9
Obese (30 +)	154	26.3
Unknown/Missing	49	8.4
*Current body size from pictogram*		
Slight (Fig. 1, 1 or 2)	64	10.9
Average (3 or 4)	189	32.3
Slightly heavy (5 or 6)	175	29.9
Heavy (7, 8, or 9)	87	14.9
Unknown	70	12.0
*Waist-to-hip ratio*		
< 0.86	180	30.77
0.86–0.93	176	30.09
> 0.93	182	31.11
Missing	47	8.03
*Height (cm)*		
< 155	168	28.7
155–159.9	170	29.1
160 +	233	39.8
Missing	14	2.4

### Measured BMI

**Fig. 2:**
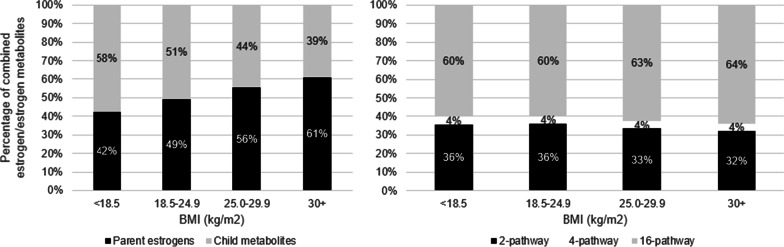
The proportion of parent estrogen concentrations increased and child pathway metabolites overall decreased (left panel) across BMI categories. When evaluating the proportion of the 2-, 4-, and 16-pathway estrogen metabolites out of the combined concentration of metabolites, 2-pathway metabolism decreased, and 16-pathway metabolism increased across increasing BMI categories (right panel)

Obese BMI (≥ 30 kg/m^2^ vs. 18.5- < 25.0 kg/m^2^) was associated with higher levels of parent estrogens (unconjugated estrone: 78.90 (66.57–93.53) vs. 50.89 (43.47–59.59), *p*-value < 0.0001; unconjugated estradiol: 27.83 (21.47–36.07) vs. 13.26 (10.37–16.95), *p*-value < 0.0001) (Fig. 2, Table 2). Positive associations between high BMI and 2-hydroxyestone, 4-hydroxyestrone, and most of the 16-alpha pathway estrogen metabolites (5 out of 7) were also observed, while associations with the 2- and 4- pathway methylated catechols were null (Table 2). After adjustment for unconjugated estradiol, the positive associations between high BMI and many of the estrogen metabolites noted did not persist but instead we observed inverse associations between higher BMI and many of the 2-pathway estrogen metabolites (including 2-hydroxyestrone, 2-hydroxyestradiol, and 2-methoxyestrone), a suggestion of an inverse association between higher BMI and 4-methoxyestrone, and a positive association between higher BMI and 16-ketoestradiol (Additional file 1: Table S1).

**Table 2:** Geometric means (pmol/L) and 95% CIs of serum estrogens/estrogen metabolites by current body mass index in postmenopausal control women not using menopausal hormone therapy in the Ghana Breast Health Study

	Geometric mean (95% CI)				p-trend	%Δ	p-diff
	Underweight (< 18.5 kg/m^2^)	Healthy weight (18.5–24.9 kg/m^2^)	Overweight (25.0–29.9 kg/m^2^)	Obese (30 + kg/m^2^)			
*N*	20	193	169	154			
Median BMI (kg/m^2^)	18	22	27	34			
Estrone	144.97 (88.25–238.13)	224.99 (170.21–297.39)	318.61 (239.30–424.21)	448.63 (336.80–597.58)	** < 0.0001**	99.4	** < 0.0001**
Unconjugated	41.70 (32.20–54.00)	50.89 (43.47–59.59)	64.64 (54.90–76.12)	78.90 (66.57–93.53)	** < 0.0001**	55.0	** < 0.0001**
Conjugated	93.85 (50.12–175.77)	165.03 (117.93–230.94)	251.15 (178.34–353.68)	366.55 (260.70–515.40)	** < 0.0001**	122.1	** < 0.0001**
Estradiol	16.77 (10.73–26.23)	19.68 (14.73–26.31)	29.63 (21.93–40.02)	41.81 (31.07–56.27)	** < 0.0001**	112.4	** < 0.0001**
Unconjugated	11.50 (8.05–16.42)	13.26 (10.37–16.95)	19.80 (15.25–25.71)	27.83 (21.47–36.07)	** < 0.0001**	109.9	** < 0.0001**
Conjugated	2.24 (0.83–6.03)	3.32 (1.65–6.67)	5.02 (2.50–10.08)	7.32 (3.67–14.59)	** < 0.0001**	120.4	** < 0.0001**
2-Hydroxyestrone	45.44 (37.43–55.16)	51.36 (45.24–58.31)	53.48 (46.66–61.30)	58.42 (51.15–66.73)	**0.01**	13.7	0.03
2-Hydroxyestradiol	12.35 (8.23–18.53)	9.38 (7.04–12.51)	8.39 (6.22–11.31)	8.50 (6.37–11.36)	0.06	− 9.4	0.24
2-Methoxyestrone	19.75 (14.41–27.06)	21.17 (17.44–25.70)	21.43 (17.55–26.17)	23.20 (18.91–28.46)	0.15	9.6	0.20
Unconjugated	8.54 (5.90–12.36)	8.56 (6.84–10.71)	9.07 (7.22–11.40)	8.42 (6.68–10.62)	0.93	− 1.6	0.84
Conjugated	8.67 (5.29–14.21)	9.57 (7.26–12.60)	9.94 (7.58–13.02)	12.75 (9.61–16.92)	**0.02**	33.3	**0.02**
2-Methoxyestradiol	10.55 (7.23–15.38)	12.92 (10.00–16.68)	12.82 (10.01–16.42)	13.17 (10.18–17.03)	0.44	1.9	0.78
Unconjugated	2.88 (2.02–4.11)	2.86 (2.27–3.60)	2.89 (2.27–3.68)	2.94 (2.32–3.73)	0.65	3.0	0.62
Conjugated	6.39 (3.61–11.29)	9.09 (6.33–13.04)	8.36 (5.88–11.89)	8.89 (6.22–12.70)	0.76	− 2.2	0.83
2-Hydroxyestrone-3-methyl ether	3.52 (2.56–4.83)	3.68 (2.97–4.58)	3.69 (2.96–4.60)	3.79 (3.06–4.71)	0.61	3.0	0.69
4-Hydroxyestrone	6.29 (4.35–9.09)	6.62 (4.76–9.21)	7.13 (5.16–9.84)	8.36 (6.06–11.52)	**0.004**	26.3	**0.007**
4-Methoxyestrone	3.95 (2.96–5.27)	3.58 (2.87–4.46)	3.62 (2.89–4.54)	3.60 (2.89–4.47)	0.85	0.5	0.94
4-Methoxyestradiol	1.48 (1.04–2.11)	1.54 (1.24–1.92)	1.45 (1.18–1.78)	1.58 (1.28–1.97)	0.73	2.6	0.71
16α-Hydroxyestrone	23.04 (14.78–35.90)	29.08 (20.92–40.42)	37.98 (27.19–53.04)	50.14 (36.11–69.62)	** < 0.0001**	72.4	** < 0.0001**
Estriol	82.19 (58.33–115.80)	82.48 (65.95–103.16)	95.37 (75.16–121.02)	105.42 (83.45–133.17)	**0.001**	27.8	**0.002**
Unconjugated	9.28 (7.62–11.29)	9.43 (8.38–10.62)	10.05 (8.97–11.27)	9.21 (8.21–10.33)	0.78	− 2.4	0.56
Conjugated	70.77 (47.79–104.79)	70.06 (54.67–89.77)	82.52 (63.50–107.24)	94.71 (73.17–122.60)	**0.0006**	35.2	**0.0007**
16-Ketoestradiol	11.97 (8.87–16.16)	17.24 (13.76–21.59)	19.72 (15.58–24.97)	27.89 (22.13–35.14)	** < 0.0001**	61.8	** < 0.0001**
16-Epiestriol	17.41 (11.22–27.02)	16.63 (13.07–21.16)	18.96 (14.91–24.09)	20.47 (16.06–26.08)	**0.011**	23.0	**0.008**
17-Epiestriol	28.50 (19.39–41.90)	25.07 (18.83–33.38)	26.02 (19.77–34.26)	27.13 (20.51–35.88)	0.44	8.2	0.27

Geometric means adjusted for age at blood draw (continuous), blood draw year (2013, 2014, 2015), smoking status (never, former, current, missing), diabetes (yes, no, missing), time since menopause (≤ 2, 3–5, 6–10, > 10, missing), ever used oral contraceptives (yes, no, missing)

*p-*trend was estimated using the Wald test for ordinal BMI category

%Δ indicates the percentage change in estrogen/estrogen metabolite levels, comparing women with current BMI ≥ 30 vs. 18.5–24.9 kg/m^2^, and was estimated by taking the ratio of the geometric mean difference in estrogen/estrogen metabolite levels between women with current BMI ≥ 30 vs. 18.5–24.9 kg/m^2^ to the geometric mean of women with current BMI 18.5–24.9 kg/m^2^, multiplied by 100

*p-*diff was estimated using the Wald test and indicates a *p-*value comparing estrogen/estrogen metabolite levels of women with current BMI ≥ 30 vs. 18.5–24.9 kg/m^2^

Bold p-values represent FDR *q*-value ≤ 0.05

### Self-reported body size

Consistent with the associations between circulating estrogens and measured BMI, women who self-reported the highest body size categories (7,8, or 9 = heaviest) had the highest estrogen levels (parent estrogens, 2-hydroxyestrone, 4-hydroxyestrone, and five of seven 16-pathway metabolites) compared with women who reported the lowest body size categories (1 or 2 = slight) (Table 3). After adjusting for unconjugated estradiol, the positive association between estrone and self-reported body size remained, while the associations with the estrogen metabolites attenuated substantially (Additional file 1: Table S2).

**Table 3:** Geometric means (pmol/L) and 95% CIs of serum estrogens/estrogen metabolites by current body size assessed using pictogram in postmenopausal control women not using menopausal hormone therapy in the Ghana Breast Health Study

	Geometric mean (95% CI)				*p*-trend	%Δ	*p*-diff
	Slight	Average	Slightly heavy	Heavy			
*N*	64	189	175	87			
Median BMI (kg/m^2^)	22	25	29	31			
Estrone	277.88 (168.63–457.91)	472.03 (299.00–745.19)	562.73 (351.76–900.24)	748.98 (449.60–1,247.71)	** < 0.0001**	58.7	** < 0.0001**
Unconjugated	52.63 (40.17–68.96)	66.56 (52.15–84.95)	74.68 (57.90–96.31)	94.03 (70.48–125.45)	** < 0.0001**	41.3	** < 0.0001**
Conjugated	189.16 (105.62–338.78)	397.12 (241.72–652.42)	474.59 (284.04–792.96)	637.85 (365.42–1,113.38)	** < 0.0001**	60.6	** < 0.0001**
Estradiol	28.47 (18.21–44.53)	41.16 (27.80–60.95)	49.45 (32.77–74.60)	66.41 (42.42–103.95)	** < 0.0001**	61.3	** < 0.0001**
Unconjugated	19.55 (13.51–28.28)	30.52 (22.13–42.09)	33.98 (24.18–47.74)	47.33 (31.93–70.18)	** < 0.0001**	55.1	** < 0.0001**
Conjugated	3.86 (1.59–9.38)	6.37 (2.83–14.32)	10.37 (4.54–23.68)	10.18 (4.16–24.94)	**0.0001**	59.8	**0.0011**
2-Hydroxyestrone	52.77 (45.52–61.18)	62.25 (55.20–70.19)	66.33 (57.88–76.03)	70.17 (58.93–83.57)	**0.0008**	12.7	**0.0005**
2-Hydroxyestradiol	12.07 (8.09–18.01)	10.33 (7.24–14.74)	10.47 (7.25–15.12)	10.17 (6.93–14.92)	0.30	− 1.6	0.18
2-Methoxyestrone	17.04 (13.44–21.59)	18.17 (14.85–22.23)	18.99 (15.31–23.57)	21.42 (16.80–27.31)	**0.02**	17.9	**0.02**
Unconjugated	6.21 (4.89–7.89)	6.58 (5.44–7.96)	6.38 (5.18–7.87)	6.79 (5.30–8.70)	0.62	3.1	0.44
Conjugated	8.59 (5.85–12.62)	10.03 (7.58–13.27)	11.61 (8.57–15.73)	14.57 (10.30–20.61)	**0.001**	45.3	**0.003**
2-Methoxyestradiol	9.77 (7.65–12.49)	10.17 (8.26–12.51)	10.40 (8.36–12.94)	9.52 (7.50–12.08)	0.83	− 6.4	0.79
Unconjugated	2.63 (2.09–3.32)	2.86 (2.38–3.45)	2.88 (2.36–3.52)	2.84 (2.29–3.52)	0.49	− 0.7	0.40
Conjugated	6.59 (4.56–9.54)	6.96 (5.23–9.27)	6.78 (4.98–9.22)	6.42 (4.59–8.97)	0.72	− 7.8	0.87
2-Hydroxyestrone-3-methyl ether	3.00 (2.32–3.89)	3.12 (2.45–3.96)	3.29 (2.56–4.24)	3.37 (2.56–4.44)	0.19	8.2	0.27
4-Hydroxyestrone	6.60 (4.51–9.65)	7.60 (5.46–10.58)	8.46 (5.96–12.00)	9.47 (6.58–13.63)	**0.0007**	24.6	**0.002**
4-Methoxyestrone	3.07 (2.52–3.74)	3.00 (2.58–3.48)	3.19 (2.72–3.76)	3.08 (2.55–3.71)	0.63	2.6	0.98
4-Methoxyestradiol	1.51 (1.16–1.95)	1.58 (1.29–1.92)	1.61 (1.29–2.00)	1.63 (1.30–2.05)	0.42	3.3	0.44
16α-Hydroxyestrone	21.42 (14.99–30.61)	30.66 (22.61–41.57)	29.25 (21.00–40.73)	37.29 (25.86–53.75)	**0.002**	21.6	**0.0001**
Estriol	85.14 (69.73–103.95)	92.92 (79.22–109.00)	95.18 (79.99–113.26)	113.48 (90.92–141.64)	**0.014**	22.1	**0.01**
Unconjugated	9.53 (8.17–11.13)	9.74 (8.64–10.98)	10.44 (9.09–11.99)	9.86 (8.50–11.44)	0.28	1.3	0.61
Conjugated	73.92 (59.01–92.59)	80.71 (67.41–96.64)	81.25 (66.65–99.06)	102.40 (80.16–130.81)	**0.017**	26.9	**0.01**
16-Ketoestradiol	11.97 (9.51–15.07)	15.74 (13.33–18.57)	15.69 (12.99–18.95)	19.92 (15.74–25.21)	**0.0002**	26.6	** < 0.0001**
16-Epiestriol	16.64 (12.80–21.63)	19.37 (15.52–24.17)	19.88 (15.86–24.92)	22.85 (17.56–29.74)	**0.008**	18.0	**0.005**
17-Epiestriol	19.86 (13.54–29.15)	20.60 (14.42–29.42)	20.95 (14.54–30.19)	20.68 (14.15–30.22)	0.70	0.4	0.71

Geometric means adjusted for age at blood draw (continuous), blood draw year (2013, 2014, 2015), smoking status (never, former, current, missing), diabetes (yes, no, missing), time since menopause (≤ 2, 3–5, 6–10, > 10, missing), ever used oral contraceptives (yes, no, missing)

*p*-trend was estimated using the Wald test for ordinal body size category

%Δ indicates the percentage change in estrogen/estrogen metabolite levels, comparing women with heavy body size category to average sized women, and was estimated by taking the ratio of the geometric mean difference in estrogen/estrogen metabolite levels between women reporting heavy body size categories minus average body size category to the geometric mean of women with average body size, multiplied by 100 (we could do this in the table to slight)

*p*-diff was estimated using the Wald test and indicates a *p*-value for comparing estrogen/estrogen metabolite levels of women with current heavy body size vs. average body size

Bold *p*-values represent FDR ≤ 0.05

### Waist-to-hip ratio

Estrone levels and many of the 16-pathway metabolites increased across increasing tertiles of WHR (WHR > 0.93 (T3) vs. < 0.86 (T1): estrone 332.58 (247.85–446.28) vs. 265.63 (196.64–358.83), *p*-trend = 0.03) (Table 4). In contrast, unconjugated 2-methoxyestrone and unconjugated 2-methoxyestradiol levels decreased across increasing tertiles of WHR [9.79 (7.81–12.26) vs. 8.36 (6.67–10.49) vs. 8.25 (6.64–10.24), 0.03; 3.20 (2.57–3.98) vs. 2.84 (2.28–3.53) vs. 2.76 (2.22–3.43), 0.02, respectively]. These associations persisted in models adjusted for unconjugated estradiol (Additional file 1: Table S3). The associations with estrone and 16-pathway metabolites were no longer apparent in models adjusted for BMI, but the inverse associations between unconjugated 2-methoxyestrone, unconjugated 2-methoxyestradiol, and estriol levels and WHR remained after adjustment for BMI (Additional file 1: Table S3).

**Table 4:** Geometric means (pmol/L) and 95% CIs of serum estrogens/estrogen metabolites by waist-to-hip ratio (WHR) tertile in postmenopausal control women not using menopausal hormone therapy in the Ghana Breast Health Study

	Geometric mean (95% CI) (model 1)			*p*-trend	%Δ
	< 0.86	0.86–0.93	> 0.93		
*N*	180	176	182		
Median BMI (kg/m^2^)	24.0	26.0	29.0		
Estrone	265.63 (196.64–358.83)	280.96 (208.05–379.43)	332.58 (247.85–446.28)	0.03	25.2
Unconjugated	59.62 (50.09–70.96)	57.50 (48.32–68.41)	65.72 (55.47–77.88)	0.18	10.2
Conjugated	195.47 (136.03–280.86)	215.62 (149.69–310.61)	261.63 (183.72–372.58)	0.02	33.8
Estradiol	25.85 (19.75–33.82)	25.68 (19.62–33.60)	30.30 (23.19–39.59)	0.16	17.2
Unconjugated	17.65 (13.75–22.66)	17.14 (13.38–21.96)	20.13 (15.63–25.92)	0.23	14
Conjugated	4.11 (2.15–7.84)	4.49 (2.34–8.61)	5.05 (2.64–9.67)	0.28	23
2-Hydroxyestrone	55.59 (48.69–63.48)	50.51 (44.35–57.51)	54.15 (47.66–61.52)	0.66	− 2.6
2-Hydroxyestradiol	8.99 (6.59–12.27)	8.83 (6.53–11.96)	9.19 (6.83–12.37)	0.78	2.3
2-Methoxyestrone	22.16 (18.06–27.20)	21.67 (17.70–26.54)	21.32 (17.62–25.80)	0.58	− 3.8
Unconjugated	9.79 (7.81–12.26)	8.36 (6.67–10.49)	8.25 (6.64–10.24)	0.03	− 15.7
Conjugated	9.40 (7.00–12.63)	10.65 (7.98–14.20)	10.96 (8.44–14.23)	0.20	16.6
2-Methoxyestradiol	12.73 (9.96–16.28)	12.52 (9.72–16.12)	12.84 (10.02–16.46)	0.89	0.9
Unconjugated	3.20 (2.57–3.98)	2.84 (2.28–3.53)	2.76 (2.22–3.43)	0.02	− 13.7
Conjugated	7.85 (5.57–11.06)	8.42 (5.92–11.99)	8.98 (6.37–12.65)	0.18	14.3
2-Hydroxyestrone-3-methyl ether	3.66 (2.94–4.57)	3.64 (2.94–4.52)	3.76 (3.05–4.64)	0.72	2.6
4-Hydroxyestrone	7.04 (5.14–9.66)	7.27 (5.25–10.06)	7.35 (5.37–10.05)	0.61	4.3
4-Methoxyestrone	3.93 (3.15–4.90)	3.41 (2.75–4.22)	3.58 (2.90–4.43)	0.14	− 8.8
4-Methoxyestradiol	1.46 (1.18–1.80)	1.51 (1.22–1.87)	1.57 (1.28–1.93)	0.26	7.6
16α-Hydroxyestrone	31.89 (22.93–44.34)	32.62 (23.35–45.57)	42.96 (31.11–59.32)	**0.003**	34.7
Estriol	82.26 (66.81–101.28)	91.05 (74.10–111.88)	102.32 (83.39–125.56)	0.01	24.4
Unconjugated	9.21 (8.28–10.25)	10.13 (9.01–11.39)	9.40 (8.47–10.43)	0.62	2
Conjugated	71.43 (56.65–90.07)	77.59 (61.76–97.49)	90.38 (72.03–113.40)	0.01	26.5
16-Ketoestradiol	17.82 (14.10–22.51)	18.56 (14.71–23.41)	22.85 (18.09–28.86)	**0.002**	28.2
16-Epiestriol	17.45 (13.81–22.04)	17.79 (14.15–22.36)	19.87 (15.80–24.98)	0.08	13.9
17-Epiestriol	25.37 (19.23–33.49)	27.06 (20.42–35.87)	26.27 (19.89–34.70)	0.63	3.5

Geometric means adjusted for age at blood draw (continuous), blood draw year (2013, 2014, 2015), smoking status (never, former, current, missing), diabetes (yes, no, missing), time since menopause (≤ 2, 3–5, 6–10, > 10, missing), ever used oral contraceptives (yes, no, missing)

*p*-trend was estimated using the Wald test for ordinal WHR category

%Δ indicates the percentage change in estrogen/estrogen metabolite levels, comparing women with highest WHR tertile to lowest WHR tertile, and was estimated by taking the ratio of the geometric mean difference in estrogen/estrogen metabolite levels between women with highest WHR tertile minus lowest WHR tertile to the geometric mean of women with lowest WHR tertile, multiplied by 100

Bold *p*-values represent FDR ≤ 0.05

### Height

There were no clear patterns of increasing or decreasing estrogen metabolism across categories of increasing measured height (Additional file 1: Table S4).

### Sensitivity analyses

Results did not change substantively after excluding women with diabetes at blood draw or those who had an underweight BMI. When considering multiple comparisons using FDR, most associations with a nominal *p*-value less than or equal to 0.01 had *q*-values less than or equal to 0.05 (as indicated with bold font in the manuscript tables).

## Discussion

In this novel cross-sectional study of circulating estrogen metabolism in postmenopausal African women, measured BMI was positively associated with higher levels of most of the measured estrogens. After adjustment for unconjugated estradiol, which showed the strongest association with BMI, positive associations between BMI and estrone as well as BMI and 16-ketoestradiol persisted, whereas BMI was inversely associated with most of the 2-pathway metabolites. Like measured BMI, self-reported body size was positively associated with higher levels of most estrogens/estrogen metabolites. Consistent with this, we observed BMI attenuated the associations between WHR and estrogens; together this suggests metabolite concentrations were driven by overall adiposity rather than fat distribution. Height was not associated with differences in estrogen metabolism.

Our findings of positive associations between current BMI and parent estrogen levels are consistent with studies conducted predominantly among White women [12]. These findings are also in line with biological evidence supporting the major source of estrogens in postmenopausal women derives from aromatization of androgens (androstenedione and testosterone) to estrogens (estriol and estradiol) in adipose tissue. To date, a limited number of studies have examined current BMI in relation to estrogen metabolism. Earlier studies using ELISA-based assays measured only two estrogen metabolites thought to be the most and the least carcinogenic: 16α-hydroxyestrone and 2-hydroxyestrone, respectively [23–26]. Results from these earlier studies supported an inverse association between adiposity and the ratio of urinary 2-hydroxyestrone to 16α-hydroxyestrone in both pre- and postmenopausal women [24, 25, 27]. In a study nested in the Prostate, Lung, Colorectal, and Ovarian Cancer Screening Trial, self-reported BMI was positively correlated with all 15 serum estrogens/estrogen metabolites among postmenopausal women [28]; however, that study did not assess whether the associations with estrogen metabolites remained after accounting for correlations with unconjugated estradiol. In a study nested in the Women’s Health Initiative Observational Study, positive associations between increasing BMI and estrogen metabolites did not remain after adjustment for unconjugated estradiol; however, consistent with the current study, inverse associations between BMI and methylated 2-catechols became apparent after adjusting for unconjugated estradiol in postmenopausal women [13]. This latter study also demonstrated that obese women appear in general to be less likely to metabolize parent estrogens into child metabolites, but more likely to favor metabolism of parent estrogens into 16-pathway estrogen metabolites over 2- or 4-pathway metabolites. Our findings corroborate these results and provide novel information about patterns of estrogen metabolism with BMI in African women.

In our study, most of the WHR-metabolite associations were not independent of BMI. In studies where body fat distribution was measured by dual-energy X-ray absorptiometry scan [29] or measured WHR [30], central obesity was not associated with circulating unconjugated estradiol independent of BMI among predominantly White postmenopausal women, suggesting that body fat distribution does not impact circulating estradiol beyond that of overall adiposity. However, the inverse associations between WHR and unconjugated 2-methoxyestrone and unconjugated 2-methoxyestradiol and positive association between WHR and estriol persisted in models additionally adjusted for BMI. This suggests that, in African women, the association between these metabolites and WHR may represent a pattern of estrogen exposure that is potentially relevant for disease risk and that warrants further exploration. As such, the independent association between these metabolites and WHR may represent a pattern of estrogen exposure that is potentially relevant for disease risk in African women and warrants further exploration.

Measured BMI and self-reported body size were associated with similar increases in parent estrogens, but only BMI measurements were related to the metabolites. The lack of signal for the metabolites with self-reported body size could be due to the imprecise nature of the pictogram or from collapsing across categories.

Current adult height in African women was not associated with differences in estrogen metabolism. This finding is consistent findings in predominantly non-Hispanic White postmenopausal women from the Women’s Health Initiative Observational Study [13].

Limitations of the current study include the use of measured circulating estrogens/estrogen metabolites at a single point in time. However, a previous study using our same assay has shown moderate to high 1-year ICCs in postmenopausal women [31], suggesting that measured serum estrogens/estrogen metabolites may also adequately represent postmenopausal levels over at least 1 year. While we used established BMI cutpoints to facilitate comparison with previous research conducted among predominantly White postmenopausal women, measures of obesity are not well established for African populations, and as such may not be as informative of disease risk.

Our study has notable strengths. Measurement error for the anthropometric measures in the current study was reduced by using measured height, weight, and waist and hip circumferences, as compared to other studies that used self-reported height/weight, etc. Other study strengths include the use of the high-performance LC–MS/MS assay that provided a comprehensive evaluation of individual estrogens/estrogen metabolites with high reliability, sensitivity, and specificity. Further, use of a large sample size limited to postmenopausal women not using hormones at blood collection and careful adjustment for potential confounders assessed at blood collection increased the validity of the results.

## Conclusions

In this comprehensive analysis of measured anthropometrics and serum estrogens/estrogen metabolites in African women, we observed strong, positive associations between measured BMI and parent estrogens in postmenopausal women. After adjustment for unconjugated estradiol, measured BMI was also associated with lower levels of 2-pathway metabolites and higher levels of 16-ketoestradiol. In studies of predominantly White women, it has been suggested that endogenous estrogen metabolism at least partially mediates the association between BMI and increased risk of postmenopausal estrogen receptor positive (ER +) breast cancer, given the observation that increasing BMI is associated with higher levels of parent estrogens and reduced concentrations of 2-pathway metabolites. The consistency of the BMI-estrogen metabolism association in this study of African women suggests that these same mechanisms may be relevant for postmenopausal ER + breast cancer risk in African women. Our data also suggest that WHR in African women may explain differences in circulating estrogen metabolite levels independent of BMI. These findings merit further evaluation in prospective studies.

## Supplementary Information

**Additional file 1:** Supplemental Tables summarizing geometric means (pmol/L) and 95% CIs of serum estrogens/estrogen metabolites by current body mass index with additional adjustment for unconjugated estradiol (Table S1); by current body size assessed using pictogram with additional adjustment for unconjugated estradiol (Table S2); by waist-to-hip ratio (WHR) tertile with additional adjustment for measured BMI (Table S3); and by height tertile (Table S4) in postmenopausal control women not using menopausal hormone therapy in the Ghana Breast Health Study.

## Data Availability

The datasets generated or analyzed for the current study are not publicly available due to data privacy of patients but are available from the corresponding author upon reasonable request.
